# Seroepidemiology of Entamoeba histolytica Infection in General Population in Rural Durango, Mexico

**DOI:** 10.14740/jocmr2131w

**Published:** 2015-04-08

**Authors:** Cosme Alvarado-Esquivel, Jesus Hernandez-Tinoco, Luis Francisco Sanchez-Anguiano

**Affiliations:** a Biomedical Research Laboratory, Faculty of Medicine and Nutrition, Juarez University of Durango State, Avenida Universidad S/N, 34000 Durango, Mexico; b Institute for Scientific Research “Dr. Roberto Rivera-Damm”, Juarez University of Durango State, Avenida Universidad S/N, 34000 Durango, Mexico

**Keywords:** *Entamoeba histolytica*, Seroepidemiologic studies, Rural population, Risk factors, Mexico

## Abstract

**Background:**

The seroepidemiology of *Entamoeba histolytica* in Mexico has been scantily studied. The aim of the study was to determine the seroprevalence and correlates of *E. histolytica* antibodies in adults in rural areas in Durango, Mexico.

**Methods:**

Through a cross-sectional study, *E. histolytica* IgG antibodies were determined in 282 adults living in rural Durango, Mexico using an enzyme-linked immunoassay. In addition, seroprevalence association with the socio-demographic, housing conditions, and behavioral characteristics of the subjects studied was investigated.

**Results:**

One hundred and eighteen (41.8%) of the 282 rural subjects had anti-*E. histolytica* IgG antibodies. Multivariate analysis showed that *E. histolytica* exposure was positively associated with source of drinking water (OR = 2.73; 95% CI: 1.33 - 5.58; P = 0.005), and poor education of the head of the family (OR = 1.53; 95% CI: 1.03 - 2.27; P = 0.03). In contrast, *E. histolytica* exposure was negatively associated with consumption of unpasteurized cow milk (OR = 0.55; 95% CI: 0.31 - 0.96; P = 0.03), and crowding at home (OR = 0.33; 95% CI: 0.17 - 0.64; P = 0.0009).

**Conclusions:**

The seroprevalence of *E. histolytica* infection found in adults in rural Durango is high compared with those reported in other Mexican populations. The correlates of *E. histolytica* seropositivity found in the present study may be useful for the planning of optimal preventive measures against *E. histolytica* infection.

## Introduction

The protozoa parasite *Entamoeba histolytica* is an important cause of morbidity and mortality worldwide [1, 2]. Infections with *E. histolytica* are common and are one of the major health problems in developing countries [3, 4]. Humans are the host of *E. histolytica* and there are no other known animal reservoirs of this parasite [5]. The clinical spectrum of *E. histolytica* infections varies from asymptomatic infection to hemorrhagic colitis and extra-intestinal disease [6]. Most persons infected with *E. histolytica* are carriers [7]. Infection with *E. histolytica* is responsible from a considerable number of cases of prolonged diarrhea in travelers [8]. In addition, infection with *E. histolytica* may lead to the development of live-threatening abscess in liver, brain [9] or lungs [5]. Transmission of *E. histolytica* occurs in areas with poor sanitation by contamination of drinking water or food with human feces [10]. Water-associated outbreaks of *E. histolytica* disease have been reported [11]. Transmission of *E. histolytica* can also be sexual [12].

Very little is known on the seroepidemiology of *E. histolytica* infection in rural adults in Mexico. Rural communities in Mexico have commonly poor sanitation, and this is an important condition for transmission of *E. histolytica* among the population. A considerable number of houses in rural Mexico have poor availability of drinkable water and poor disposal of excretes. Therefore, contamination of water and food with *E. histolytica* is highly feasible to occur in rural communities. The lack of laboratory tests for diagnosis of *E. histolytica* infection in rural health centers does not allow having reliable statistical information about the magnitude of *E. histolytica* exposure in rural Mexico. We sought to determine the seroprevalence of *E. histolytica* IgG antibodies in adults in rural Durango, Mexico. Furthermore, socio-demographic and behavioral characteristics of the rural subjects associated with *E. histolytica* seropositivity were investigated.

## Materials and Methods

### Study design and study population

The design of this study was cross-sectional. We analyzed stored serum samples used in a previous survey about the seroepidemiology of *Toxoplasma gondii* infection in rural populations in Durango, Mexico [13]. Serum samples were collected from December 2006 to August 2007 in three rural communities: San Dimas, Villa Montemorelos, and Santa Clara. Inclusion criteria for enrollment were: 1) inhabitants of rural Durango; 2) aged 18 years and older; 3) any sex; and 4) who accepted to participate in the survey. Exclusion criteria for enrollment were: 1) subjects with insufficient amount of serum; and 2) subjects with incomplete socio-demographic and behavioral data. Selection of subjects was performed randomly. In total, 282 subjects were included in this study, 94 of them were inhabitants of San Dimas; 82 were inhabitants of Villa Montemorelos, and 106 were inhabitants of Santa Clara.

### General socio-demographic and behavioral characteristics of rural adults

Socio-demographic and behavioral characteristics of the participants were obtained with the aid of a standardized questionnaire. Socio-demographic items included age, birthplace, residence, educational level, socio-economic status, and employment. Housing conditions of the participants were determined by using the Bronfman’s criteria [14]. This tool allowed to assess crowding, type of flooring (ceramic, concrete, soil), availability of drinkable water (within the house, out of the house), and form of elimination of excretes (flush toilet, latrine, or other). In addition, the educational level (years of education) of the head of the family was recorded. Behavioral items included consumption of unpasteurized milk or untreated water, consumption of unwashed raw vegetables or fruits, frequency of eating away from home (in restaurants or fast food outlets), raising farm animals, foreign travel, and contact with soil (gardening or agriculture).

### Laboratory tests

Serum samples of the participants were analyzed for anti-*E. histolytica* IgG antibodies by a commercially available enzyme immunoassay “*E. histolytica* IgG (Amebiasis) ELISA” kit (Diagnostic Automation Inc., Calabasas, CA). All assays were performed following the manufacturer’s instructions. Samples were run along with positive and negative controls in each assay. According to the information included in the kit’s insert, the enzyme immunoassay used has a sensitivity of 92% and a specificity of 100%.

### Statistical analysis

We used the software Epi Info version 7 and SPSS version 15.0 to perform the statistical analysis. For calculation of the sample size, a reference seroprevalence of 4.49% [15] as the expected frequency for the factor under study, 300,000 as the population size from which the sample was selected, 2.5% confidence limits, and a 95% confidence level (CI) were considered. The result of the sample size calculation was 263 subjects. The Pearson’s Chi-squared test and the Fisher exact test (when values were small) were used for initial comparison of frequencies among groups. Socio-demographic characteristics, housing conditions, and behavioral variables with a P value equal to or less than 0.05 obtained in the bivariate analysis were further analyzed by multivariate analysis to determine their association with *E. histolytica* seropositivity. Odds ratios (OR) and 95% CIs were calculated by using logistic regression analysis with the Enter method. The Hosmer-Lemeshow goodness of fit test was used to assess the fitness of our regression model. Statistical significance was set at P value < 0.05.

### Ethical aspects

Only archival serum samples and data from a previous study [13] were used in the present study. The ethical committee of the Mexican Social Security Institute in Durango City, Mexico approved this previous survey. The purpose and procedures of the study were explained to all participants, and a written informed consent was obtained from all of them.

## Results

Most participants were female (78.0%), of low socioeconomic status (77.3%), and unemployed (76.6%). Mean age of participants was 42.91 ± 17.53 years old (range 18 - 91 years). One hundred and eighteen (41.8%) of the 282 rural subjects had anti-*E. histolytica* IgG antibodies. A correlation of *E. histolytica* seropositivity and socio-demographic and behavioral characteristics and housing conditions of the rural subjects studied is shown in Table 1. Of the socio-demographic data, housing conditions, and behavioral characteristics assessed, the variables age, community of residence, education, consumption of unpasteurized milk, source of drinking water, crowding at home, and educational level of the head of the family had P values < 0.05 by bivariate analysis. Other socio-demographic data, housing conditions, and behavioral characteristics including occupation, socio-economic status, type of flooring at home, form of elimination of excretes, foreign travel, raising animals, consumption of untreated water, unwashed raw vegetables or fruits, eating away from home and contact with soil had P values > 0.05 by bivariate analysis. Further analysis using logistic regression of the socio-demographic, housing conditions, and behavioral characteristics of rural adults showed that *E. histolytica* exposure was positively associated with source of drinking water (OR = 2.73; 95% CI: 1.33 - 5.58; P = 0.005), and poor education of the head of the family (OR = 1.53; 95% CI: 1.03 - 2.27; P = 0.03) (Table 2). In contrast, logistic regression analysis showed that *E. histolytica* exposure was negatively associated with consumption of unpasteurized cow milk (OR = 0.55; 95% CI: 0.31 - 0.96; P = 0.03), and crowding at home (OR = 0.33; 95% CI: 0.17 - 0.64; P = 0.0009). The result of the Hosmer-Lemeshow test (P = 0.60) suggested a good fit of our regression model.

**Table 1:** Bivariate Analysis of a Selection of Exposure Variables and Seroprevalence of *E. histolytica* in General Population in Rural Durango

Characteristic	No. of subjects tested	Positive ELISA results		Odds ratio	95% confidence interval	P value
		No.	%			
Gender						
Male	62	27	43.5	1.1	0.61 - 1.93	0.75
Female	220	91	41.4	1.0		
Age groups (years)						
30 or less	75	23	30.7	1.0		
31 - 50	120	47	39.2	1.5	0.78 - 2.68	0.22
> 50	87	48	55.2	2.8	1.45 - 5.31	0.001
Community						
One	94	14	14.9	1.0		
Two	82	52	63.4	9.9	4.80 - 20.43	< 0.0001
Three	106	52	49.1	5.5	2.77 - 10.90	< 0.0001
Educational level						
No education	27	17	63.0	2.6	1.14 - 5.88	0.01
Education	255	101	40.4	1		
Occupation						
Employed^a^	66	29	43.9	1.1	0.64 - 1.95	0.69
Unemployed^b^	216	89	41.2	1.0		
Socio-economic level						
Low	218	97	44.5	1.6	0.91 - 2.95	0.09
Medium	64	21	32.8	1.0		
Traveled abroad						
Yes	44	21	47.7	1.3	0.69 - 2.53	0.38
No	238	97	40.8	1.0		
Unpasteurized cow milk consumption						
Yes	182	68	37.4	0.6	0.36 - 0.97	0.03
No	100	50	50	1.0		
Unwashed raw vegetables						
Yes	51	19	37.3	0.8	0.42 - 1.47	0.46
No	231	99	42.9	1.0		
Unwashed raw fruits						
Yes	66	25	37.9	0.8	0.45 - 1.41	0.45
No	216	93	43.1	1.0		
Untreated water						
Yes	139	59	42.4	1.1	0.65 - 1.68	0.83
No	143	59	41.3	1.0		
Soil contact						
Yes	250	108	43.2	1.7	0.76 - 3.68	0.19
No	32	10	31.3	1.0		
Source of drinking water						
Home	172	93	54.1	4.0	2.33 - 6.85	< 0.0001
Out of home	110	25	22.7	1.0		
Sewage disposal						
Pipes	130	60	46.2	1.0		
Latrine, other	152	58	38.2	0.7	0.44 - 1.15	0.17
Crowding						
No	62	38	61.3	1.0		
Yes	220	80	36.4	0.4	0.20 - 0.64	0.0004
Education of the head of family						
7 or more years	50	15	30	1.0		
4 - 6 years	119	45	37.8	1.4	0.69 - 2.88	0.33
Up to 3 years	113	58	51.3	2.5	1.21 - 4.99	0.01
Floor at home						
Ceramic	19	7	36.8	1.0		
Concrete	184	69	37.5	1.0	0.38 - 2.73	0.95
Soil	79	42	53.2	1.9	0.69 - 5.45	0.20

^a^Employed: agriculture, business, construction worker, factory worker, professional, other. ^b^Unemployed: housewives, students or none occupation.

**Table 2:** Results of the Multivariate Regression Analysis

Variable	P value	Odds ratio	95% confidence interval
Age	0.25	1.24	0.85 - 1.82
Community	0.13	1.37	0.90 - 2.08
No education	0.37	1.54	0.58 - 4.04
Consumption of raw cow milk	0.03	0.55	0.31 - 0.96
Water at home	0.005	2.73	1.33 - 5.58
Crowding	0.0009	0.33	0.17 - 0.64
Education of the head of family	0.03	1.53	1.03 - 2.27

## Discussion

The seroepidemiology of *E. histolytica* infection in rural Mexico has been scantily studied. Although amebiasis has been recognized as a major health problem in Mexico for many years [16, 17], very little is known about the seroprevalence of *E. histolytica* infection and risk factors associated with this infection in Mexican populations. Therefore, this study was performed to know the frequency of *E. histolytica* exposure among adults living in rural areas in the northern Mexican state of Durango. We found an overall 41.8% seroprevalence of *E. histolytica* infection in adult people of the three rural communities studied. This *E. histolytica* seroprevalence is higher than other *E. histolytica* seroprevalences in Mexican populations reported so far. In a previous study in people living in northern Mexican states, researchers found a low (< 5%) seroprevalence of *E. histolytica* in all states surveyed [18]. Nearly 25 years have passed between these studies and difference in the seroprevalences might suggest an increase in *E. histolytica* exposure. However, different laboratory methods were used among the studies; in the previous study, a homemade ELISA was used, whereas we used a commercially available ELISA. The sensitivity and specificity of the homemade ELISA were 95% and 90.7%, respectively [18]. According to the manufacturer of the commercially available ELISA used in the present study, the assay has a sensitivity and specificity of 92% and 100%, respectively. The seroprevalence found in adults in rural Durango is also higher than the mean 8.41% seroprevalence in 32 federal entities and ≤ 8% in northern states reported in a national survey [17]. However, the presence of antibodies against *E. histolytica* in the national survey was detected by an indirect hemagglutination test, which is also a different method from the one we used. The seroprevalence of *E. histolytica* found in our study is also higher than the 4.49% seroprevalence reported in a second national seroepidemiology survey of *E. histolytica* infection by using an ELISA [15]. The seroprevalence found in rural Durango is also higher than the 13.8% prevalence of *E. histolytica* infection in a rural community in the central Mexican state of Morelos obtained by polymerase chain reaction in stools [19]. However, comparison of the seroprevalence of *E. histolytica* infection with the prevalence of infection based on polymerase chain reaction in stools should be interpreted with care since a poor correlation between intestinal infection and anti-amebic antibody levels has been reported [19]. It is not clear why subjects in rural Durango have a much higher seroprevalence of *E. histolytica* exposure than other populations in Mexico. We searched for potential risk factors associated with *E. histolytica* in rural Durango. Multivariate analysis showed that *E. histolytica* exposure was positively associated with source of drinking water and poor education of the head of the family. Subjects with water supply within their home had a higher seroprevalence of *E. histolytica* infection than subjects who obtained water from outside their homes. This finding suggests that *E. histolytica* infection was acquired at home by drinking contaminated water from the public water supplying systems. In Mexico, water supplied by pipes to houses from public water wells is not fully potable. However, many people drink such water in spite of the risk for acquiring infectious diseases. This wrong practice may reflect poor education. In fact, *E. histolytica* exposure was associated with poor education of the head of the family in our study. In contrast, the negative associations of *E. histolytica* exposure with consumption of unpasteurized cow milk and crowding at home found in the present study suggest that these characteristics did not play any important role in *E. histolytica* infection among the subjects studied.

### Conclusions

We concluded that the seroprevalence of infection with *E. histolytica* found in rural populations in Durango is higher than *E. histolytica* seroprevalences reported in other Mexican populations. The correlates of *E. histolytica* seropositivity found in the present study can be used for an optimal planning of preventive measures against *E. histolytica* infection.
